# Psychiatric Disorders in Patients with Chronic Obstructive Pulmonary Disease: Clinical Significance and Treatment Strategies

**DOI:** 10.3390/jcm13216418

**Published:** 2024-10-26

**Authors:** Pasquale Moretta, Nicola Davide Cavallo, Claudio Candia, Anna Lanzillo, Giuseppina Marcuccio, Gabriella Santangelo, Laura Marcuccio, Pasquale Ambrosino, Mauro Maniscalco

**Affiliations:** 1 Istituti Clinici Scientifici Maugeri IRCCS, Neurorehabilitation Unit of Telese Terme Institute, 82037 Telese Terme, Italy; nicoladavide.cavallo@unicampania.it (N.D.C.); laura.marcuccio@icsmaugeri.it (L.M.); 2 Department of Psychology, Università della Campania ‘Luigi Vanvitelli’, 81100 Caserta, Italy; gabriella.santangelo@unicampania.it; 3 Istituti Clinici Scientifici Maugeri IRCCS, Pulmonary Rehabilitation Unit of Telese Terme Institute, 82037 Telese Terme, Italy; claudio.candia@icsmaugeri.it (C.C.); anna.lanzillo@icsmaugeri.it (A.L.); giuseppina.marcuccio@icsmaugeri.it (G.M.); mauro.maniscalco@icsmaugeri.it (M.M.); 4 Istituti Clinici Scientifici Maugeri IRCCS, Scientific Directorate of Telese Terme Institute, 82037 Telese Terme, Italy; pasquale.ambrosino@icsmaugeri.it

**Keywords:** psychiatric disorders, anxiety, depression, COPD, rehabilitation

## Abstract

Chronic obstructive pulmonary disease (COPD) is a debilitating lung disease characterized by chronic airflow limitation and persistent respiratory symptoms. It is a major cause of morbidity and mortality worldwide, significantly impacting healthcare systems with considerable socioeconomic consequences. The main risk factors include exposure to tobacco smoke, environmental pollutants, occupational dust, and genetic factors such as alpha-1 antitrypsin deficiency. COPD is often associated with extrapulmonary comorbidities, particularly psychiatric disorders like depression and anxiety, which worsen patients’ quality of life and prognosis. The prevalence of these disorders in patients with COPD varies significantly but is always higher than in healthy controls. Psychiatric disorders can negatively influence COPD management and vice versa, creating a complex bidirectional interaction. Depression and anxiety are the most common psychiatric comorbidities in patients with COPD, influenced by factors such as social isolation, physical limitations, and fear of exacerbations. Diagnosis of these psychiatric disorders is complicated by the overlap of symptoms with those of COPD. However, some screening tools can facilitate early detection. The treatment of psychiatric disorders in patients with COPD in a rehabilitation setting requires a multidisciplinary approach involving pulmonologists, neurologists and psychologists. Pharmacological therapy with antidepressants shows mixed results in terms of efficacy and safety, with some studies reporting benefits on quality of life and others suggesting an increased risk of exacerbations and pneumonia. An integrated approach that includes the assessment and intervention of mental disorders is essential to improve the overall management of COPD and the quality of life of patients. This narrative review provides an overview of the main psychiatric comorbidities in COPD patients, aiming to fill gaps in the literature and suggest areas for future research.

## 1. Introduction

Chronic obstructive pulmonary disease (COPD) is a widespread, debilitating, and heterogeneous lung disease characterized by chronic and usually progressive airflow limitation and persistent respiratory symptoms [1]. It is a leading cause of morbidity and mortality worldwide [2] and represents a major challenge to global health systems, with significant socioeconomic and health consequences [3,4]. COPD is the third leading cause of death worldwide [5], and it is estimated to affect over 380 million people globally [2], with a prevalence ranging from 7.38% to 12.64% among individuals over 40 years old [6]. The primary risk factor for COPD is prolonged exposure to tobacco smoke, in both active and passive ways [7]. However, other factors, such as environmental pollutants, occupational dust, and chemicals, may also contribute significantly. Additionally, genetic factors, including alpha-1 antitrypsin deficiency, can predispose individuals to develop COPD [8].

COPD is a complex disorder, marked by systemic inflammation in addition to local pulmonary inflammation that may negatively affect various organic systems. Patients with COPD often have extrapulmonary manifestations and comorbidities that may impact the quality of life and are associated with worse prognosis and outcomes, such as increased hospital admissions, reduced response to treatment, and higher mortality rates [9]. Among these comorbidities, psychiatric disorders are particularly relevant in patients with COPD, significantly impacting their quality of life and disease management [10,11]. Studies have shown that the prevalence of psychiatric disorders in COPD is very variable, ranging from 12.1 to 33% [9,12]. However, all data consistently show that psychiatric comorbidities are significantly more common in patients with COPD than in healthy controls [12,13]. This convergence of evidence has led the Global Initiative for Chronic Obstructive Lung Disease to include a recommendation for assessing psychiatric conditions in newly diagnosed patients with COPD [14].

Nevertheless, the relationship between psychiatric disorders and COPD appears to be very complex and intricate. Psychiatric disorders are described both as comorbid conditions and as independent modifiers of disease progression, demonstrating a close relationship of mutual influence in which the roles of cause and effect are often nuanced and difficult to establish [15,16,17].

The primary objective of this narrative review is to provide a comprehensive overview of the main psychiatric comorbidities in patients with COPD. Specifically, the review aims to provide insights into clinical significance and interpretation by attempting to fill current gaps in the literature and suggesting areas for future research to improve the understanding and management of psychiatric disorders in COPD. Moreover, this narrative review aims to highlight the importance of a holistic approach to COPD management that integrates mental health assessment and intervention, ultimately improving the quality of life of patients suffering from this chronic disease.

## 2. Overview of Common Psychiatric Disorders in COPD

COPD is frequently accompanied by a spectrum of psychiatric disorders that significantly impact patients’ overall well-being [18]. Among these, depression and anxiety are the most commonly reported, probably because they are more easily investigated and recognized by non-mental health professionals (e.g., pulmonologists). This has led to a large body of evidence regarding the relationship between depression and anxiety and COPD [10,11,16] while neglecting other psychiatric disorders that may be comorbid with COPD [19,20]. Other psychiatric conditions may also be prevalent in COPD, such as substance abuse disorder and severe mental illness, including bipolar disorder and schizophrenia [21,22]. In contrast, the co-occurrence between COPD and post-traumatic stress disorder (PTSD) is still very unclear, and this relationship should be clarified by future research [19]. Understanding the range of psychiatric comorbidities in patients with COPD is crucial for comprehensive treatment and improving patient outcomes.

As reported above, depression is one of the most prevalent psychiatric disorders in patients with COPD. The chronic nature of COPD, with its associated fatigue, physical limitations, and social isolation, contributes significantly to the development of depressive symptoms [23,24]. On the other hand, depression increases social isolation and physical inactivity, significantly affects patients’ quality of life, and is associated with poorer clinical outcomes, including increased hospitalizations and mortality, and a diminished quality of life [25]. Symptoms such as persistent sadness, lack of interest in activities, and fatigue can overlap with COPD symptoms, complicating the diagnosis and management of both conditions.

Prevalence estimates vary widely, partly due to the diverse measurement tools used and the varying degrees of illness severity across studies. Studies have found approximately 24–40% of patients with COPD suffer from clinical depression, which is markedly higher than the prevalence in the general population [13,25,26]. In addition, patients with COPD are 1.9 times more likely to commit suicide than people without COPD [27].

Anxiety disorders are also highly prevalent among patients with COPD, with panic disorder, generalized anxiety disorder, and specific phobias being the most common [28]. Anxiety can exacerbate COPD symptoms, leading to increased breathlessness, avoidance behaviors, and reduced physical activities and social interactions, which in turn can worsen overall health status [29]. Conversely, the experience of breathlessness and fear of exacerbations can trigger or worsen anxiety symptoms. Patients with anxiety are more likely to report higher levels of dyspnea and fatigue, contributing to a vicious cycle of anxiety and physical discomfort [25]. Anticipation of dyspnea in patients with COPD may be associated with increased physiological fear responses, activating fear-related areas of the brain such as the insula, anterior cingulate cortex, and amygdala. This reflects anticipatory fear in patients, leading to activity avoidance and further increases in breathlessness [30].

Anxiety disorders are present in approximately 10–55% of patients with COPD [25,31], and these wide rates are potentially due to difficulties in differentiating between symptoms of COPD and symptoms of anxiety [32]. In addition, the reported prevalence of specific anxiety disorders varied widely: generalized anxiety disorder (6–33%), panic disorder (with and without agoraphobia) (0–41%), specific phobia (10–27%), and social phobia (5–11%) [31].

Among the psychiatric disorders found in patients with COPD, substance abuse disorders are also pervasive [12,22,33]. Given the severe impact of COPD on patients and the high prevalence of psychiatric disorders, substance use may be a way for patients to alleviate symptoms of anxiety and depression [34,35]. Furthermore, among substance abuse disorders, cannabis abuse has the highest frequency [12]. This result could be explained by the fact that most patients with COPD have a positive history of smoking, and many of them are still dependent on tobacco substitutes. A summary of the esteemed prevalence rates has been reported in Table 1.

Finally, evidence suggests a strong link between severe mental illness (i.e., schizophrenia, bipolar disorder) and COPD. People diagnosed with bipolar disorder or schizophrenia may have a higher risk of developing COPD compared to the general population [36]. However, the exact reason for this link is not yet known. Factors such as poor living conditions and extensive smoking over a lifetime are possible explanations, but additional research is needed to support this assertion. However, COPD might be an independent risk factor for the development of bipolar disorder [20,21]. Future studies should thoroughly investigate deeply these relationships.

### Risk Factors for Psychiatric Disease in COPD

The predictors of psychiatric disorders in COPD, particularly with regard to anxiety and depression, include various demographic and clinical factors. Age, gender, lower economic status, and COPD severity significantly influence the prevalence of these psychiatric conditions [21,37]. For example, women with COPD are more susceptible to depression and anxiety compared to men and experience higher levels of dyspnea and worse quality of life without significant differences in lung function [38]. Additionally, anxiety and depression are more prevalent in younger patients with COPD (under 60 years) [39,40], while older age is a predictor of depression among caregivers of patients with COPD [41].

Other significant factors associated with depression in patients with COPD include BMI, dyspnea, history of cardiovascular disease, and current smoking status [42,43]. Moreover, a lower education level is a risk factor for both depressive and anxious symptoms in these patients [44]. The severity of COPD is directly related to the risk of developing psychiatric comorbidities [45,46]. Patients with severe COPD are more likely to experience depression and anxiety, probably due to the increased physical limitations and dependence on others for daily activities [39].

Additionally, systemic inflammation and long-term use of corticosteroids and other medications (i.e., short-acting beta-agonists) can have psychiatric side effects [20,47,48]. In this regard, corticosteroids such as prednisone and budesonide are commonly prescribed for managing COPD exacerbations due to their anti-inflammatory effects. However, their use has been widely linked to significant psychiatric side effects. In fact, long-term use of systemic corticosteroids can lead to mood disorders such as depression, anxiety, and mania, as well as cognitive impairments and psychosis in more severe cases, with higher doses increasing the risk [49]. Mood and cognitive changes appear to be common with corticosteroids. However, these side effects may be mild and reversible [50]. Short- and long-acting beta-agonists are frequently used for quick relief of bronchospasm. However, these drugs are known to cause side effects such as nervousness, tremors, dizziness, and an increased heart rate, all of which can exacerbate psychiatric symptoms, in particular anxiety, and adversely influence the perception of COPD symptoms [20,51]. Finally, anticholinergics, including tiotropium and ipratropium, are commonly used in COPD management for their bronchodilatory effects. While generally well-tolerated, they can cause confusion, hallucinations, and cognitive impairment, particularly in older adults [52]. A study by Coupland et al. [53] found that exposure to several types of anticholinergic drugs is associated with an increased risk of dementia.

In summary, clinicians need to be aware of the potential psychiatric side effects of COPD medications. Careful monitoring of psychiatric symptoms in patients receiving these treatments can improve overall patient outcomes, particularly in those already prone to mental health disorders.

In conclusion, several factors serve as important clinical predictors of anxiety, depression, and overall psychiatric disorders in patients with COPD [40]. Comprehensive management of COPD should include regular screening and treatment of psychiatric comorbidities to enhance overall patient health and well-being.

## 3. Bidirectional Relationship Between COPD and Psychiatric Disorders

The relationship between COPD and psychiatric disorders is intricate, likely stemming from a complex interplay of physiological, behavioral, and psychosocial factors [54]. Moreover, emerging evidence suggests a bidirectional influence, with COPD affecting psychiatric conditions and vice versa [17].

Pre-existing psychiatric disorders, such as depression or anxiety, may contribute or exacerbate COPD symptoms and negatively impact disease management [55,56].

In some cases, psychiatric disorders may precede and contribute to the development or worsening of COPD course [57]. Depression and anxiety, for example, can lead to negative behaviors such as smoking, poor adherence to treatments, and a sedentary lifestyle, which are significant risk factors for the onset and progression of COPD [15,17]. Smoking, in particular, is a major cause of COPD and is often linked to mental health issues [58]. Individuals with psychiatric conditions may use smoking as a form of self-medication and are more likely to be cigarette smokers than those without psychiatric comorbidities, inadvertently increasing their risk of developing COPD [59]. In addition, the association between depression and anxiety with impaired cognitive function in patients with COPD can lead to distorted thoughts and beliefs, potentially causing patients to misinterpret symptoms such as breathlessness [60]. Furthermore, there is some overlap in symptoms between COPD and psychiatric illnesses. Dyspnea is a hallmark symptom of COPD and is also frequently observed in individuals with psychiatric disorders, particularly anxiety disorders. For example, during a panic attack, individuals may experience a rapid increase in breathing rate, leading to a sensation of breathlessness. This can create a vicious cycle where the fear of dyspnea exacerbates anxiety, further increasing the perception of breathlessness [61]. On the other hand, according to the cognitive interpretation of emotions [62], dyspnea in COPD could be interpreted in the context of anxiety symptoms and, therefore, further increase the feeling of breathlessness and worsen COPD symptoms. In this sense, anxiety may increase COPD symptoms, and COPD symptoms may increase anxiety symptoms in a dysfunctional reciprocity.

Moreover, the chronic, progressive, and debilitating nature of COPD significantly impacts the patient’s quality of life [63]. The physical limitations, frequent exacerbations, and hospitalizations associated with COPD can lead to psychological distress, thereby increasing the risk of developing psychiatric disorders [64,65]. The ongoing struggle with breathlessness, fatigue, and physical debilitation often results in feelings of helplessness, frustration, and despair, which can trigger or exacerbate depression and anxiety [66]. Additionally, depression and anxiety may lead to social isolation, non-adherence to treatments, and avoidance of physician consultations, which may further increase mental health issues and impact the treatment, resulting in more severity and rapid progression of COPD [37]. An alternative hypothesis is that common risk factors such as smoking, poor socioeconomic status, and systemic inflammation may independently contribute to the development of both COPD and psychiatric disorders, indicating a co-occurrence rather than a direct causal relationship [17,67]. This model highlights that while COPD and psychiatric disorders may influence each other, they can also exist as comorbidities due to overlapping aetiological factors [68,69].

### 3.1. Etiology and Pathophysiological Mechanisms Linking COPD and Psychiatric Disorders

Understanding the link between COPD and psychiatric disorders is critical for comprehensive patient care. The exact mechanisms underlying the connection between psychiatric disorders and COPD remain uncertain. However, several pathophysiological pathways are proposed to explain the association between these conditions [60,70,71].

The interaction of biological, psychological, and social factors may play a role in the etiology of psychiatric disorders in COPD.

Systemic inflammation may be a key link between these conditions. Chronic inflammation, a hallmark of COPD, can lead to neuroinflammation, which has been implicated in the pathogenesis of psychiatric disorders [70,72,73]. COPD might increase the risk of central nervous system abnormalities, including psychiatric disorders, by increasing systemic inflammation [20]. Dysregulation in cytokine signaling might lead to the occurrence of mood and anxiety diseases and cognitive dysfunction [74]. The excessive or prolonged production of proinflammatory cytokines can result in chronic inflammation and conditions such as anxiety or mood disorders [74,75]. Furthermore, oxidative stress, another key feature of COPD, results from an imbalance between free radicals and antioxidants in the body. Increased oxidative stress markers, such as protein carbonyls and reactive oxygen species (ROS), are commonly observed in patients with COPD, indicating extensive oxidative damage [76]. Moreover, oxidative stress has been implicated in the pathogenesis of depression [77]. Depressed patients often exhibit altered antioxidant defenses and elevated levels of oxidative markers, such as malondialdehyde (MDA) and protein carbonyl content (PCC). These oxidative imbalances are thought to contribute to the neuroinflammation and neurodegeneration observed in depression [71].

Furthermore, dyspnea, hypoxia, and hypoxemia can also have a role in the development of symptoms of mood disorders and anxiety in COPD [78]. In particular, chronic hypoxia has been linked to oxidative stress, neuronal damage, and neuroinflammation [79], with subsequent psychiatric manifestations such as depressive and anxiety symptoms in COPD patients [20]. This link between hypoxia and psychiatric disorders seems to depend on several different but complementary mechanisms, including (1) the reduction of brain oxygen-dependent processes and, in particular, of the activity of tryptophan hydroxylase, which is the rate-limiting enzyme in the synthesis of serotonin; (2) the synthesis of oxytocin and vasopressin, whose receptors are expressed in the amygdala and the bed nucleus of the stria terminalis, areas linked to anxiety and fear; (3) the alteration of mitochondrial dynamics; and (4) a direct action on peripheral chemoreceptors, determining a strong activation of neuronal circuits linked to anxiety and panic [80]. Intermittent chronic hypoxia can cause oxidative stress and neuroinflammation [74], which can lead to psychiatric manifestations [20].

Another possible psychophysiological mechanism includes the autonomic nervous system and immune dysregulation. Chronic stress can lead to persistent activation of the sympathetic nervous system (SNS) and may also increase the risk for exacerbations in patients with COPD via autonomic pathways [60]. Similarly, chronic stress due to COPD, together with depression and anxiety, could compromise immune responses, leading to an increased risk of exacerbations of COPD symptomology [60,81].

Furthermore, the systemic effects of COPD, such as decreased physical activity and social isolation due to breathlessness, can exacerbate psychiatric symptoms. Reduced physical activity is associated with an increased risk of depression, while social isolation can lead to feelings of loneliness, anxiety, and depression [17,25]. Besides, the stigma associated with COPD, often perceived as a self-inflicted disease due to smoking, can further exacerbate these feelings and reduce the likelihood of seeking help [82].

Finally, medications used to manage COPD can also contribute to psychiatric symptoms. For example, corticosteroids and H1-blockers, commonly used in COPD treatment, have been associated with developing a mental disorder, particularly at higher doses [48,83]. Additionally, psychiatric drugs such as antipsychotics may be related to an increased risk of smoking, thus contributing to the pathogenesis of COPD indirectly [84].

In conclusion, current evidence suggests that individuals with COPD who also experience comorbid psychiatric disorders often experience more severe symptoms and poorer outcomes [65,66]. This may be due to different cognitive, behavioral, and social factors, and psychophysiological mechanisms, which overall highlight the need to improve the recognition and treatment of psychiatric disorders in subjects with COPD.

**Figure 1:**
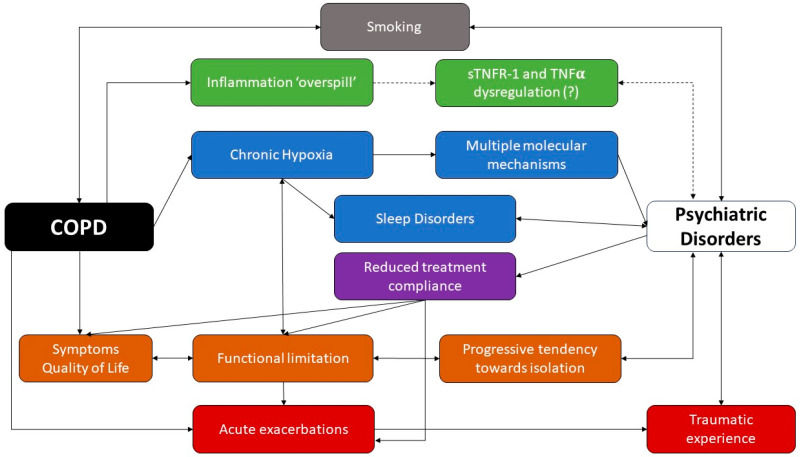
COPD and psychiatric disorders: a complex liaison. While smoking seems to be a relevant risk factor for both conditions, the chronic local inflammation observed in COPD patients might occasionally undergo a ‘spillover’, with an increased systemic circulation of some inflammatory cytokines and chemokines. Chronic hypoxia has been linked to neuronal and hormonal alterations through multiple mechanisms, while sleep disorders have been linked to intermittent hypoxia, which is extremely common among psychiatric patients. Patients with COPD tend to experience isolation and limitations due to their impaired quality of life. Moreover, COPD patients may undergo acute exacerbations, which contribute to the worsening of the quality of life and to the progression toward disability; however, sometimes, they might be perceived as traumatic experiences. Finally, psychiatric disorders have been linked to a reduced compliance with pharmacological treatment, which impairs the patient’s quality of life and might be a risk factor for acute exacerbations. Full arrows show established relationships between items, while dashed arrows have been used to show unclear/uncertain relationships.

A schematic representation of the complex interactions between COPD and psychiatric disorders has been proposed in Figure 1.

### 3.2. Impact of Psychiatric Disorders on COPD Outcomes

Psychiatric comorbidities seem to affect several domains of COPD patients, from quality of life to exacerbation rate and mortality, thus contributing to a further worsening of the patients’ perspectives.

In particular, an interesting systematic review and metanalysis by Blakemore et al. [10], including six studies, highlighted how psychiatric disorders anxiety and depression are significantly associated with worsening levels of HRQoL at a one year follow-up (pooled r = 0.48, 95% CI 0.37–0.57, P,0.001; pooled r = 0.36, 95% CI 0.23–0.48, P,0.001 for depression and anxiety, respectively); however, only three studies concerning depression and two concerning anxiety were eligible for the metanalysis, thus limiting the calculation of the effect size.

In a study conducted in Spain [85] including a population of 512 COPD patients monitored for two years, the presence of depression was associated with a 128% increase in the risk of acute exacerbations (unadjusted OR 2.28, 95% CI 1.17–4.12). However, after adjusting for relevant risk factors linked to exacerbations, the association lost statistical significance.

Finally, a metanalysis involving 16 studies and a total of 28,759 COPD patients underscored how depression and anxiety are linked to an increased risk of mortality among COPD patients, with a relative risk (RR) of 1.83 (95% CI: 1.00–3.36) for depression and of 1.27 (95% CI: 1.02–1.58) for anxiety disturbs [16].

## 4. Assessment and Diagnosis of Main Psychiatric Disorders in COPD

Psychiatric disorders in COPD are often underdiagnosed, and this can negatively affect patient outcomes [66,86]. Therefore, they should be identified and managed more effectively in clinical practice. However, diagnosing psychiatric disorders in patients with COPD presents several challenges. Symptoms of depression and anxiety, such as fatigue, sleep disturbances, and loss of appetite, can overlap with those of COPD, making it difficult to distinguish between the conditions. Additionally, stigma and reluctance to discuss mental health issues may prevent patients from reporting their symptoms [24].

The gold standard for diagnosing these conditions is a clinical interview conducted by a mental health clinician. However, screening instruments, such as questionnaires, are valuable in identifying patients who would benefit from such an interview. The Severe Respiratory Insufficiency (SRI) questionnaire [87] is an example of a screening tool used to evaluate disease severity in individuals with COPD and other respiratory disorders, focusing specifically on assessing anxiety among other parameters.

Other validated screening diagnostic tools, such as the Anxiety Inventory for Respiratory Disease (AIR), which is a brief, self-administered tool for screening and measuring anxiety in patients with COPD [88]. The COPD Anxiety Questionnaire (CAF) is another reliable tool for the early identification of COPD-related anxiety, offering a straightforward approach to recognizing anxiety symptoms specific to this patient population [89]. The Primary Care Evaluation of Mental Disorders Patient Questionnaire (PRIME-MD PQ) detects the five most common psychiatric disorders, including depression and anxiety [90]. The Generalized Anxiety Disorder seven-item scale (GAD-7) efficiently identifies and scores common anxiety symptoms and is useful for both screening and severity assessment of generalized anxiety disorder in clinical practice [91]. Furthermore, the General Health Questionnaire-version 20 (GHQ-20) can screen for psychological distress in patients with COPD [92]. Additionally, the Beck Anxiety Inventory [93,94], the Hamilton Anxiety Rating Scale [95], and the State-Trait Anxiety Inventory [96] exclusively measure anxiety symptoms. Other questionnaires, such as the Beck Depression Inventory (BDI), are extensively used to evaluate depression [97].

Additionally, there are several screening tools to detect psychiatric disorders in COPD, such as the Hospital Anxiety and Depression Scale (HADS) [98] and the Patient Health Questionnaire (PHQ-9) [99], are effective for identifying symptoms of anxiety and depression. These tools are simple, quick to administer, and suitable for primary and specialized care [25]. All the mentioned clinical tools are summarized in Table 2.

### Multidisciplinary Teams: Role of Primary Care, Pulmonologists and Psychologists

The comprehensive management of psychiatric disorders in patients with COPD requires the involvement of multidisciplinary teams comprising pulmonologists, primary care physicians, psychologists, psychiatrists, neurologists, and respiratory therapists. This collaborative approach ensures holistic care, addressing both the physiological and psychological dimensions of the condition.

Primary care physicians and pulmonologists play a pivotal role in the early identification and management of psychiatric disorders in patients with COPD. They are often the first point of contact and may screen for psychiatric symptoms during routine consultations and refer to mental health professionals when necessary. However, detecting mood disorders in this setting remains a significant challenge [17]. Therefore, the role of psychologists becomes extremely useful, contributing significantly to the assessment and management of psychiatric disorders in patients with COPD, including providing interventions such as cognitive behavioral therapy (CBT), which is effective in reducing symptoms of depression and anxiety [100]. They also offer support for behavioral changes, including smoking cessation, which is crucial for COPD management [101].

Therefore, it is crucial to integrate psychiatric care into the standard respiratory assessment and treatment protocols of patients with COPD. Recognizing the reciprocal relationship between psychiatric symptoms and COPD and understanding the negative prognostic implications of these concurrent symptoms are pivotal in guiding physician practice. Early diagnosis and appropriate intervention can positively influence the prognosis of COPD.

## 5. Treatment Strategies for Psychiatric Disorders in COPD

The importance of treating psychiatric disorders in patients with respiratory diseases has been underlined in the guidelines for pulmonary rehabilitation [14].

Over the past 30 years, pharmacological treatment of psychiatric disorders in patients with COPD has been extensively studied, with sometimes conflicting evidence regarding its safety and efficacy, particularly in relation to the risk of acute exacerbations, pneumonia, and infections [102].

Antidepressants of all classes have been linked to an increase in the risk of acute exacerbations, hospitalization, and pneumonia in patients with COPD [103,104]. However, the impact of antidepressants on mild and moderate exacerbations of COPD remains controversial, with one study [104] describing a reduction of such events in the first 90 days following an antidepressant prescription. The mechanisms linking the use of antidepressants and the development of pneumonia in patients with COPD are still unclear, but it has been hypothesized that pneumonia might be a consequence of the drugs’ side effects [103]. In contrast to the above, however, it has been demonstrated that treatment with sertraline, a selective serotonin reuptake inhibitor (SSRI), has a positive impact on quality of life and symptom control in patients with COPD diagnosed with depression, with a reduction in the COPD Assessment Test score [105]. Nonetheless, the same authors report that no effect on lung function was observed during the treatment with sertraline; the absence of effects on lung function was then confirmed by more recent evidence [106]. Nonetheless, the positive effect of sertraline and other SSRIs might be explained by the evidence that patients with COPD diagnosed with depression tend to have lower compliance to their inhaled therapy in comparison to the non-depressed ones [107]. Conversely, when such patients are treated with antidepressants, their compliance with inhaled medications improves [108].

Tricyclic antidepressants (TCAs) have also been used in COPD patients for the treatment of depression [109], but their role in clinical practice is limited by the reported elevated incidence of side effects, mainly cardiovascular and anticholinergic [110]. However, a relatively recent Cochrane systematic review and metanalysis [111] concluded that the trials exploring the pharmacological effects of SSRI and TCAs for the treatment of depression and anxiety among COPD are mostly heterogeneous, provide low-quality evidence, and are largely inconclusive. In this scenario, novel and specifically designed trials should be designed.

In light of the above, the impact of antidepressant drugs on patients with COPD seems still unclear, with studies demonstrating both benefits such as the improvement of symptom control and quality of life, as well as impactful side effects like pneumonia and severe COPD exacerbations, especially in the first three months of treatment. Therefore, clinicians should use antidepressants in patients with COPD only after a thorough evaluation of the potential risks and benefits deriving from such treatment [111].

SSRIs should be preferred to TCAs at least for the first line of treatment, given the relatively lower rate of reported side effects [110].

While evidence on antidepressant drugs is somewhat contrasting, hypnotic and anxiolytic agents have been shown to present with impactful negative effects on the respiratory system [112,113,114], and, therefore, their use in patients with COPD with sleep disorders or anxiety should be carefully evaluated [115]. Attempts at demonstrating an improvement in dyspnea and exercise endurance with anxiolytic treatment have failed [116,117]. Furthermore, benzodiazepine treatment in patients with COPD has been linked to a higher incidence of acute respiratory failure [118] and an all-cause mortality rate [119].

Finally, data on the use of antipsychotic drugs for managing schizophrenia or bipolar disorder in patients with COPD are quite limited [110]. However, the treatment with antipsychotic drugs in patients with COPD seems to induce a higher rate of acute respiratory failure [120], as well as a higher rate of bloodstream infections [121], possibly due to an intrinsic immunomodulatory effect [122]. The mechanisms of such events, however, are still largely unclear, and further studies seem to be needed in order to achieve sounder answers.

Although pharmacotherapy has been fundamental as a treatment strategy for anxiety and depression [31], it has been demonstrated that psychotherapy, particularly cognitive behavioral therapy (CBT), has provided a significant reduction in anxious and depressive symptoms in patients who have undertaken the psychotherapeutic process, compared to those who followed only pharmacological therapy [100,123]. Several studies reported that symptoms of anxiety and depression improved significantly in the CBT group compared to the control group, and most of the changes lasted over time and had a beneficial impact on self-perceived quality of life [100,124,125]. Another technique used in the treatment of psychological symptoms in COPD, belonging to CBT, is the so-called “Mindfulness” (MBCT) [126]. The purpose of MBCT is to increase awareness and non-judgmental attention toward current internal and external experiences through meditation exercises. It has been shown that MBCT can reduce symptoms of chronic diseases and improve the management and well-being of patients with COPD [126]. A phenomenological study verified that MBCT can benefit patients with asthma and COPD suffering from anxiety and depression according to the following qualitative parameters given, including the combination of pulmonary rehabilitation advice with some mental attitudes, such as awareness; greater acceptance and reduction of stigma linked to the disease; a new relational development between breathing, activities and related thoughts; the notice of subtle physical sensations and the first signs of difficulty breathing; be creative with limitations and remove mental barriers to become more active; have a stronger sense of control [127,128].

The impact of the interventions for depression among COPD patients has been explored in a metanalysis, which included 29 randomized controlled trials and a total of 2063 COPD patients [129] and its update [130]. The authors demonstrated that the pooled effects of psychological and/or lifestyle interventions led to small but significant reductions in symptoms of depression, with a standardized mean difference (SMD) of 0.28 (95% CI: −0.41, −0.14), and anxiety (SMD −0.23; 95% CI: −0.38, −0.09). When stratifying the studies according to the intervention components, multidisciplinary pulmonary rehabilitation was the only intervention associated with significant improvements in symptoms of depression (SMD −0.47; 95% CI: −0.66, −0.28) and anxiety (SMD −0.45; 95% CI: −0.71, −0.18). Conversely, cognitive and behavioral treatment approaches as well as relaxation techniques were associated only with numerical reductions in psychiatric symptoms, while no effect was demonstrated for self-management interventions, including disease education. These data were further corroborated by analyzing the five trials that included both psychological and exercise components: the effect size increased to 0.64 for depression and to 0.59 for anxiety, thus suggesting that multidisciplinary interventions might be more effective rather than single-domain interventions.

## 6. Future Directions in Research and Clinical Practice

The complex relationship between COPD and psychiatric disorders requires continued research and innovation in both diagnostic and therapeutic approaches. The future perspectives in managing psychiatric disorders in patients with COPD can be grouped into several key areas. First, the development and implementation of integrated care models that combine respiratory and mental health care can significantly improve patient outcomes. These models would involve multidisciplinary teams working collaboratively to address the holistic needs of patients with COPD, including their psychiatric comorbidities. From this perspective, telemedicine approaches and digital health platforms can facilitate the integration of mental health services into routine COPD care, especially in remote or underserved areas. Second, advancements in personalized medicine could lead to more tailored treatments for patients with COPD and psychiatric disorders. In this regard, by understanding the genetic, molecular, and environmental factors contributing to both COPD and psychiatric conditions, clinicians can develop individualized treatment plans. Certainly, targeted interventions could be guided through the identification of patients at higher risk for psychiatric comorbidities by means of biomarkers for inflammation, oxidative stress, and other pathophysiological mechanisms. Furthermore, longitudinal studies and clinical trials are essential to better understand the mechanisms linking COPD and psychiatric disorders.

Another important point concerns the therapeutic options. While current pharmacological treatments for psychiatric disorders in patients with COPD have shown mixed results, ongoing research into new medications and treatment regimens holds promise. This includes novel antidepressants and anxiolytics with fewer respiratory side effects and better safety profiles; more specific anti-inflammatory and antioxidant therapies, targeting the systemic inflammation and oxidative stress that link COPD and psychiatric disorders; exploring the potential therapeutic beneficial effect of cannabinoids and psychoactive drugs for both psychiatric and respiratory symptoms, given their emerging evidence in other chronic conditions [131]. On the side of non-pharmacological treatments, psychotherapeutic interventions, especially cognitive behavioral therapy (CBT), have proven effective in managing anxiety and depression in patients with COPD. Future research should also focus on designing tailored psychotherapy programs specifically for patients with COPD, addressing the unique challenges posed by chronic respiratory disease. Moreover, it could be useful to explore the effectiveness and feasibility of digital CBT, expanding access through online platforms and mobile apps. Another crucial field of intervention concerns lifestyle and behavioral modifications. Future possible directions include the enhancements of smoking cessation programs with the integration of mindfulness and stress-reduction techniques by incorporating practices like yoga and meditation to reduce anxiety and improve overall well-being.

Last, the early detection of psychiatric disorders in patients with COPD is of paramount importance to reduce their impact on the course of respiratory symptoms and quality of life. This could be achieved through the implementation of routine screening protocol in care settings and by using machine learning and big data approaches to identify patients at risk for developing psychiatric comorbidities.

## 7. Conclusions

The future of managing psychiatric disorders in patients with COPD lies in a comprehensive, integrated approach that addresses both the physical and psychological aspects of the disease. By leveraging advancements in personalized medicine, pharmacotherapy, psychotherapeutic interventions, lifestyle modifications, and early detection, healthcare providers can significantly improve the quality of life for patients with COPD. Ongoing research and innovation will be key to overcoming the challenges and complexities of this dual burden, ultimately leading to better patient outcomes and reduced healthcare costs.

## Figures and Tables

**Table 1:** Prevalence of psychiatric disorders among COPD patients. Abbreviations: COPD, chronic obstructive pulmonary disease.

Reference	Study Type	Disease	Subjects (n)	Reported Prevalence
[12]	Case-control study	Generalized anxiety disorder	COPD = 74 Controls = 74	5.41% 1.35%
		Major depressive episode	COPD = 74 Controls = 74	2.70% 0
		Panic disorder	COPD = 74 Controls = 74	4.05% 0
		Substance dependence	COPD = 74 Controls = 74	16.21% 1.35%
[26]	Metanalysis	Depression	COPD = 5552 Controls = 5211	27.1% [25.9–28.3] * 10.0% [9.2–10.8] *
[25]	Review	Depression and anxiety	-	Depression: 10–42% Anxiety: 10–19%
[33]	Observational study	Depression	Chronic bronchitis = 1440 Emphysema = 518 Controls = 32,509	8.7% (6.8–10.7) ^§^ 7.5% (4.7–10.2) 3.6% (3.3–3.8)
		Bipolar disorder	Chronic bronchitis = 1440 Emphysema = 518 Controls = 32,509	3.3% (2.1–4.6) - 0.8% (0.7–0.9)
		Panic disorder	Chronic bronchitis = 1440 Emphysema = 518 Controls = 32,509	6.5% (4.6–8.3) - 1.8% (1.6–2.0)
		Social phobia	Chronic bronchitis = 1440 Emphysema = 518 Controls = 32,509	7.2% (5.3–9.1) 6.5% (3.7–9.2) 2.8% (2.5–3.0)
		Substance dependence	Chronic bronchitis = 1440 Emphysema = 518 Controls = 32,509	7.9% (5.0–10.8) - 2.8% (2.6–3.0)
[31]	Metanalysis	Generalized anxiety disorder	COPD inpatients = 358 COPD outpatients = 333	10–55% 13–46%

* Data expressed as mean and 95% confidence interval. ^§^ Data expressed as relative percentage and 95% confidence interval.

**Table 2:** Clinical tools adopted in the assessment of psychiatric disorders in COPD.

Clinical Scales	Clinical Function and Psychometric Structure	Authors
Severe Respiratory Insufficiency (SRI)	A specific measure of health-related quality of life in patients receiving home mechanical ventilation. The scale is provided by a five-point Likert scale, a scaling method that measures either positive or negative responses. Scores ranging between 0 and 100. Higher scores are attributed to better health-related quality of life (HRQL).	[87]
Anxiety Inventory for Respiratory Disease (AIR)	A scale to screen and measure anxiety in patients with COPD. The score range is from 0 to 30, and the high scores indicate elevated symptoms of anxiety.	[88]
COPD Anxiety Questionnaire (CAF)	CAF is a self-report scale developed to measure specifically anxiety in patients with COPD. Each item is rated by the patients themselves using a five-point Likert scale ranging from “never” (scored 0) to “always” (scored 4), with a total score ranging from 0 to 100. Higher scores indicate high levels of anxiety.	[89]
Primary Care Evaluation of Mental Disorders Patient Questionnaire (PRIME-MD PQ)	The PRIME-MD PQ is a self-administered one-page questionnaire consisting of 26 yes/no questions about the presence of symptoms and signs during the past month.	[90]
Generalized Anxiety Disorder seven-item scale (GAD-7)	This seven-item scale (GAD-7) is used to screen for anxiety or to measure its severity. Although designed as a screening tool for generalized anxiety, the GAD-7 also performs reasonably well as a screening tool for three other common anxiety disorders—panic disorder, social anxiety disorder, and post-traumatic stress disorder. GAD-7 total score for the seven items ranges from 0 to 21. Scores of 5, 10, and 15 represent cut points for mild, moderate, and severe anxiety, respectively.	[91]
General Health Questionnaire-version 20 (GHQ-20)	It is a self-administered screening scale for identifying minor psychiatric disorders. It consists of 20 items asking respondents to compare their current status with their normal situation. Answers are scored on a four-point Likert-type scale ranging from 0 (less than usual) to 3 (much more than usual). Scores can range from 0 to 60, and lower scores means better mental health.	[92]
Beck Anxiety Inventory (BAI)	A self-administered scale consisting of a list of 21 items that measures how much the patient has been bothered by that symptom during the past month. Scores ranging from 0 to 63: minimal anxiety levels (0–7), mild anxiety (8–15), moderate anxiety (16–25), and severe anxiety (26–63).	[93,94]
Hamilton Anxiety Rating Scale (HARS)	The scale consists of 14 items, each defined by a series of symptoms, and measures both psychic anxiety (mental agitation and psychological distress) and somatic anxiety (physical complaints related to anxiety). A total score ranging from 0–56: scores <17 indicates mild severity; between 18–24 indicates mild to moderate severity; between 25–30 indicates moderate to severe.	[95]
State—Trait Anxiety Inventory (STAI)	It is a 40-item self-report measure of anxiety using a four-point Likert-type scale (from 0 to 3 points) for each item. It has two scales: State anxiety, i.e., how one feels at the moment; and Trait anxiety, i.e., how one generally feels. It is composed of 20 items to be rated on a 1–4 scale, with higher scores meaning higher levels of anxiety; the cut-off score for the presence of relevant anxiety symptoms is 40.	[96]
Beck Depression Inventory (BDI)	It is a 21-item, multiple-choice inventory. Respondents are asked to rate each item based on four response choices according to the severity of the symptoms, ranging from the absence of a symptom to an intense level, during the past week. Each question is scored on a four-point scale ranging from no impairment (0) to severe impairment (3). The maximum score is 63; a cut-off score indicative of mild depressive symptoms is higher than 10, and for severe depressive symptoms the cut-off score is higher than 30.	[97]
Hospital Anxiety and Depression Scale (HADS)	It is a 14-item self-report scale for symptoms of depression and anxiety in a general medical population of patients. Each item is scored on a scale ranging from 0 (no symptom) to 3 (severe symptom). Scores ranging from 8–10 indicate doubtful cases, while scores ≥ 11 indicate clinically relevant cases. A cut-off score ≥ 8 can be considered optimal for both sensitivity and specificity for the diagnosis of clinically relevant anxiety and depression.	[98]
Patient Health Questionnaire (PHQ-9)	It is a multipurpose instrument for screening, diagnosing, monitoring, and measuring the severity of depression. Scores range from 0 to 27, where high scores mean greater presence of depressive symptoms.	[99]

## Data Availability

Not applicable.
